# Gratitude despite unease among Swedish male forensic psychiatric patients with substance use disorders: an interview study

**DOI:** 10.1080/17482631.2024.2418671

**Published:** 2024-11-02

**Authors:** Charlotta Svensson, Malin Hildebrand Karlén, Eirini Alexiou, Sepideh Olausson, Christopher Holmberg

**Affiliations:** a Department of Forensic Psychiatry, Sahlgrenska University Hospital, Gothenburg, Gunnilse, Sweden; b Institute of Health and Care Sciences, University of Gothenburg, Gothenburg, Göteborg, Sweden; c Centre for Ethics, Law and Mental Health (CELAM), Department of Psychiatry and Neurochemistry, Institute of Neuroscience and Physiology, Sahlgrenska Academy, University of Gothenburg, Gothenburg, Gunnilse, Sweden; d Department of Psychology, University of Gothenburg, Gothenburg, Göteborg, Sweden; e Department of Anaesthesiology and Intensive Care, Sahlgrenska University Hospital, Gothenburg, Göteborg, Sweden; f Department of Psychotic Disorders, Sahlgrenska University Hospital, Gothenburg, Mölndal, Sweden

**Keywords:** Comorbidity, forensic nursing, forensic psychiatric care, qualitative content analysis, rehabilitation, substance use disorder

## Abstract

**Purpose:**

Forensic psychiatric inpatient care in Sweden is characterized by strict regulations and adherence to court-mandated rules, with a median care time of 7 years. Comorbity has significant clinical implications, impacting health and well-being, violent behaviour and criminal recidivism, and prolonging inpatient stays. This study investigated the individual experiences and perspectives of Swedish patients in forensic psychiatric care with substance use (SU) disorders, focusing on past, present and future orientations.

**Methods:**

Ten male forensic psychiatric inpatients were individually interviewed following a semi-structured interview guide. A qualitative content analysis was conducted to inductively analyse the participants’ experiences. The study adhered to the Standards for Reporting Qualitative Research.

**Results:**

Under the main theme of “embracing harmony in opposition”, three categories emerged: (1) unveiling the beginning of SU and its impact on navigating challenges in care, (2) exploring current confined care and gratitude despite unease and (3) yearning for a somewhat normal life.

**Conclusions:**

Participants’ subjective perspectives underscored the importance of factors such as forensic nurses/healthcare providers in forensic psychiatry considering the patients’ life narratives, their aspirations for the future, their need for a secure environment and patient—staff relationships. These findings highlight the need for structural interventions that emphasize the importance of tailored and comprehensive health plans within rehabilitation.

## Introduction

In Sweden, substance use (SU) care in forensic psychiatric settings is not prioritized in relation to the extent of the problem (Green et al., 2023). In recent years, the number of patients with substance use disorders (SUDs) in forensic psychiatric care (FPC) has increased not only in Sweden, but also worldwide (Penney et al., 2019; Swedish National Forensic Psychiatric Register, 2022). Nevertheless, evidence of the effects and guidelines for SUD treatment in combination with comorbidity problems remains limited. Active treatment for SUDs is essential in FPC, given its association with elevated mortality rates, violent behaviour, prolonged treatments and criminal recidivism (Durbeej et al., 2015; Ojansuu et al., 2019; Pickard & Fazel, 2013; Sivak et al., 2023). SUDs also influence patients’ ability to rehabilitate and successfully progress through forensic psychiatric systems (McFadden et al., 2022). For these reasons, addressing SUD care is clinically significant for the dual purpose of FPC: providing evidence-based treatment for patients and contributing to societal protection by rehabilitating offenders sentenced to care and ultimately facilitating their reintegration into society. To effectively mitigate SUDs in forensic psychiatric settings, it is crucial to explore the firsthand experiences of the patients involved.

## Forensic psychiatric care in Sweden

FPC is for individuals who have been legally convicted of a criminal offence while under the influence of a severe mental illness. In Sweden, FPC is regulated under the umbrella of involuntary care, as stipulated by the Swedish Forensic Mental Care Act (1991:1129). According to the Swedish Penal Code, there is no mental impairment defence, individuals with severe mental illnesses are responsible for their criminal actions. As of 2022, Sweden had approximately 3,292 patients admitted to FPC, and of those, 1,164 were treated as outpatients (Swedish Association of Local Authorities and Regions, 2022), often combined with special court supervision. Sentences to FPC are of unlimited time, being evaluated every six months, with a median duration of 7 years (Sivak et al., 2023).

FPC patients generally have complex needs and often suffer from low psychosocial functioning. As a result, their most common living condition before being convicted is homelessness. Many participants had prior experiences with jail, institutional care, caring for young persons (i.e., special provisions), and voluntary or coercive psychiatric care before their present stay in FPC. Most (84%) FPC patients in Sweden are male, the population’s median age is 39 and the most common primary diagnosis (63%) according to the International Classification of Diseases, Tenth Revision (ICD-10) is a psychotic disorder (i.e., F20–F29). Comorbidity is common, and most patients (65%) have comorbidity with an SUD. A large proportion of the cohort has also been convicted of violent crime, such as homicide, acts of bodily harm or threats (Degl’innocenti et al., 2021). Along with these trends, the risk factors for criminal recidivism related to SUDs in Sweden have shifted in recent years. Using forensic psychiatric register-based data, researchers found that both violent convictions and criminal recidivism were more often associated with SU in later years (Alexiou et al., 2023). The patient group has therefore been called “triply troubled”, as they suffer from psychiatric problems, SUDs and violent behaviour (Lindqvist, 2007).

## Integrated substance use disorder care

According to the Swedish National Forensic Psychiatric Register (2022), which covers 85% of Sweden’s population in FPC, patients with SUDs in FPC often undergo drug screening (85%), whereas proactive and comprehensive treatments, such as medication (25%) and psychoeducation (34%), including motivational interviewing and relapse prevention, remain rare. These trends are troubling, given research that suggests that FPC patients need such interventions as well as integrated models of care that combine SU rehabilitation and mental health interventions (Durbeej et al., 2015; Eagle et al., 2019). In general, integrated care is characterized by healthcare service strategies that promote coordination across levels of care, thereby enhancing the outcomes of care (Gröne & Garcia-Barbero, 2001). Schaefer et al. (2011) have argued that one reason why integrated care for SUDs and psychiatric disorders might be lacking is the misconception that SUDs can be sufficiently addressed by requiring patients to abstain from substances for an extended period, even though they may continue to grapple with psychological cravings. Another possible reason is the organizational division in Sweden between healthcare, which is administered by regional health authorities, and social services, which are organized by municipalities (Matscheck & Piuva, 2020). SUD care, however, usually occurs at the intersection between healthcare and social services; whereas healthcare treats physical abstinence and medical problems and provides psychological treatment, social services not only focus on social problems, but also provide certain kinds of psychosocial treatment programmes. Therefore, a more integrated model of SUD care is needed. Implementing new structures requires resources, however, and it is estimated that Sweden has spent 100 times less on SUD prevention than what the societal costs truly are, according to the Swedish Government Official Reports, We can do better! Knowledge-based drugs policy focused on life and health (SOU 2023:62).

As indicated by Green et al. (2023), FPC patients experience that staff display a penalizing attitude towards SU relapses instead of adopting an explorative attitude in follow-up communications with patients (i.e., to identify and clarify the source of SU and to work together to prevent future relapses). These authors also reported a dilemma expressed by staff regarding their dual role of being responsible for treatment, risk management and motivating patients to stay drug-free, yet post-relapse, of punishing them for regression or stagnation in their treatment by, for example, impeding their daily activities. Perspectives on individualized care emphasizing the unique characteristics of patients have been recommended as an evidence-based practice to improve the quality of SU care and treatment (Marchand et al., 2019). Indeed, it is crucial to consider the potential impact of rehabilitation philosophies that prioritize the patient’s perspective and involvement in treatment, including person-centred care (PCC). Two of the major principles in the PCC philosophy and approach are the patient’s narrative and the patient—professional partnership (Ekman & Swedberg, 2022). For this reason, it is essential to attempt to understand patients’ narratives, including listening to their experiences, in order to be able to build a trustworthy partnership. This necessity motivates further exploration of patients’ experiences in this context to be able to outline what they consider important.

## Aims

In our study, we aimed to explore the experiences and past-, present- and future-oriented perspectives of Swedish FPC patients regarding SU and SUD care. With this in mind, the following research questions guided the study:

1. How do participants describe their experiences with SU and SUD care before being admitted to FPC?
2. How do participants experience their current SUD care in FPC?
3. What motivators and barriers do participants experience in relation to SUD care and life after discharge?

## Materials and methods

### Design

We employed a qualitative explorative study design, chosen for its suitability in facilitating in-depth explorations of patients’ experiences with and perspectives on their care (Hunter et al., 2019). Individual interviews were selected for data collection to enable participants to express and elaborate on their subjective perspectives, understandings, and experiences. The Standards for Reporting Qualitative Research (O’Brien et al., 2014) were used to properly report key elements of the study (see the Supplementary Material).

### Sampling

A purposive sampling strategy was employed (Polit & Beck, 2021). Patients diagnosed with SUDs and deemed capable of providing informed consent, as evaluated by the psychiatrists responsible for them, were invited to participate. Exclusion criteria were having an acute psychiatric condition (e.g., manic episodes or psychotic impairment) or an imminent risk of violence, as assessed by the unit’s psychiatrist. Written information about the study (e.g., posters) was displayed in common areas within the units, and prospective participants were invited to an information meeting with the interviewer, during which they received details about the study and could ask questions.

The first author (i.e., CS) individually approached 20 patients to participate, while an unknown number (i.e., approx. 9) were invited in a group setting by a research assistant. Ultimately, 10 patients total chose to participate. Reasons for not participating included feeling too tired, already being engaged in another research project, and feeling that participation was not personally relevant.

### Participants

All participants were male and 25–51 years old, with a median age of 35 years. They were all under the care of the Forensic Mental Care Act (1991:1129) due to a mental illness severe enough to justify sentencing to FPC, which most often includes having conditions such as psychotic disorders or overall low psychosocial functioning. We categorized the patients’ disorders according to the Hierarchical Taxonomy of Psychopathology (HiTOP; Kotov et al., 2017), a new research classification of mental illness that aims to overcome the shortcomings of traditional classification systems (e.g., *Diagnostic and Statistical Manual of Mental Disorders* and *the International Classification of Diseases*). HiTOP combines co-occurring diagnosis into spectra and dimensions to overcome problems such as diagnostic instability, comorbidities, and boundary problems. The psychiatric problem areas were self-reported by participants to gain an overview of each participant’s range of psychiatric problem areas; those areas were reported in the form of HiTOP spectra to protect anonymity, for the diagnostic combinations in the sample were highly specific and risks compromising patients’ anonymity. All 10 participants had an SUD, a psychiatric problem area within HiTOP’s disinhibited externalizing spectrum. The most common comorbidity was a comorbidity on the thought disorder spectrum (*n* = 4); other comorbidities were thought disorders, either in combination with internalizing (*n* = 3) or with additional disinhibited externalizing (*n* = 2) diagnoses apart from SUD. One participant described only problems on the antagonistic externalizing spectrum. In addition, some of the participants had intellectual disabilities and/or autism (*n* = 3), which are not specifically represented in HiTOP. The median duration in FPC was 4 years (range = 1–16 years). Participants were recruited from four units that treat patients with similar diagnoses and safety levels but that vary in psychosocial functioning. All participants except one completed the Extended Alcohol Use Disorder Identification Test (AUDIT-E) (Babor & Robaina, 2016) and the Extended Drug Use Disorder Identification Test (DUDIT-E) (Berman et al., 2007). Those self-reported assessments showed that most participants were polydrug users of substances such as amphetamines, cocaine, opiates (e.g., heroin), Tetrahydrocannabinol (e.g., cannabis products), sedative drugs (e.g., benzodiazepines), and painkillers (e.g., synthetic opioids such as tramadol). Approximately half of participants reported a high motivation for treatment, and the other half reported a moderate level of motivation, while one reported not being motivated for treatment at all. Most participants reported consuming alcohol rarely or never, whereas only two reported severe, problematic alcohol consumption.

### Data collection

The individual interviews adhered to a semi-structured interview guide (Kallio et al., 2016; see Supplementary Materials) comprising nine open-ended questions. Those questions, clarified or adapted to suit each participant’s context, were organized around themes reflecting the three mentioned research questions. The guide also included suggestions for follow-up questions (e.g., “Can you elaborate?” and “Can you provide an example?”) if participants answered ambiguously or superficially. Regarding the second research question, participants were also prompted with questions from the Substance Use Paralleling behaviour Framework (SUPBF) to initiate a conversation about their psychological abstinence behaviour. That framework is designed to help staff to identify behaviours indicative of substance dependence in controlled environments (Schaefer et al., 2011). One participant did not want to answer SUPBF, and two did not answer any of the questions.

The interviews were conducted in a designated room at the participants’ wards between June 2022 and February 2023 and lasted 25–90 min (median = 32 min). The interviews were audio-recorded and transcribed verbatim by the first author. The transcribed data were securely stored while following robust measures to safeguard participants’ confidentiality and prevent unauthorized access.

### Data analysis

The transcribed interviews were subjected to qualitative content analysis following an inductive approach, as described by Graneheim and Lundman (2004) and Graneheim et al. (2017). The unit of analysis was thus the transcribed interview, and the meaning units reflected the aim and research questions. In analysis, the transcribed interviews were first read several times to identify meaning units (i.e., words, sentences, or paragraphs) related to each other in content (Graneheim & Lundman, 2004). Second, the meaning units were condensed if they were too extensive but without affecting their core meaning by abstracting it on a higher logical level, and thereafter codes were created. Third, the codes were given labels reflecting the study’s aim and research questions and to describe the meaning units in relation to the context. Fourth, the codes were sorted into subcategories describing the manifest content in the text—that is, what was expressed on the surface level. Fifth and finally, we grouped the manifest content of the text in categories built on condensed units, codes, and subcategories. See Table 1. Although qualitative content analysis has been described as a linear process, the actual procedure involved going back and forth between the whole text and its parts while constantly considering and reassessing individual parts of the text in relation to the whole. To ensure credibility, we employed the strategy of investigator triangulation. One of us (i.e., CH) independently analysed three interview transcripts to compare the results with those of the interviewer (i.e., CS; Korstjens & Moser, 2018). Furthermore, the final versions of the categories underwent extensive discussion among all authors (i.e., CS, CH, MHK, SO, and EA) until consensus was reached.

**Table 1.** Examples from the qualitative content analysis process.

Meaning unit	Code	Subcategory	Category
Get finances in order, find housing, and such. I don’t have a place to live. Find new friends. I’ve cut ties with everything. Need to start over from scratch. Take new steps in life, towards a new life. Not mix with bad company again (7)	A new life	Staying motivated to change one’s circumstances to lead a normal life	Yearning for a somewhat normal life
“Now I have really… I have changed a lot. I have made some missteps, but everyone can make mistakes if you have problem with drugs. But right now, I feel training, food… a bit of medicine, family…. So, I’m not looking forward to taking drugs, really… (4)”	Changed a lot	Challenges and support-related needs to promote recovery	Yearning for a somewhat normal life

### Ethics approval and consent to participate

Our study was conducted in consideration of the principles of the Declaration of Helsinki (World Medical, 2013) and approved by the Swedish Ethical Review Authority (No. 2022 -00,671-01).

Studying individuals in custody and receiving care in incarceration places an ethical demand on researchers to be sensitive and ensure ethical principles during each step of their research. In our study, all participants received information both orally and in writing that detailed the voluntary nature of their participation and their right to withdraw from participating at any time for any or no reason without experiencing any impact on the continuity or duration of their care. It should be noted that the participants were subject to coercive conditions that potentially impinged upon their unfettered decision-making about participation. Participants were also apprised of the confidentiality measures in place, including the assignment of pseudonyms (i.e., participant codes) in lieu of their actual names. The signing of informed consent was a requisite for all participants to participate in the study. The interviewer reminded participants before their interviews that they were free to not answer questions that made them uncomfortable.

### Reflexivity

Reflexivity is anchored in the subjective personal, interpersonal, methodological and contextual factors that influence the research process and form the research into being (Olmos-Vega et al., 2022).

This inductive qualitative content analytical study was conducted in a medium-security FPC facility in western Sweden. The patients were cared for within four locked rehabilitation wards with modern and customized architecture. There were 18 patients who were cared for by one nurse and at least four other care staff per ward and shift. Nurses in Swedish FPC can either be registered nurses or mental health nurses, which is an extensive qualification. There is a lack of focus on SU/SUD in their education, so knowledge of the subject depends on individual interests or former clinical experience.

The interviewer, CS, who works as a mental health nurse at the clinic with 15 years of clinical experience (forensic psychiatry care and SU care), informed the participants about her separate roles as a doctoral student and a clinical nurse. In addition, no patients were asked to participate for whom the interviewer was primarily responsible in terms of care or treatment plans. Throughout the process, the findings were also critically examined in consideration of the researchers’ interdisciplinary preunderstandings, given their clinical and research-related knowledge in the field (i.e., mental health nurses, a psychologist, forensic psychiatrist and nurse).

## Results

### Overarching theme: embracing harmony in opposition

The overarching theme captured discrepancies and contrasts in the participants’ experiences with their situation. This reflected participants’ dual view on being coerced into receiving care, their future views on SU and discrepancies in patients’ and staff members’ views on diagnosis. By exploring the duality in such perspectives, there is the potential to find a balance, despite the opposing elements. A common standpoint was striving for normality, which needs to be explored and tailored to the individual, also acknowledging that opposing viewpoints or feelings often coexist or vary over time and show the complexity of human emotions and thoughts.

The analysis of the interviews resulted in three major categories: (1) unveiling the beginning of SU and its impact on navigating challenges in care, (2) exploring current confined care and gratitude despite unease and (3) yearning for a somewhat normal life, as shown in Table 2.

**Table 2.** Overview of categories and subcategories.

Categories	Subcategories
Unveiling the beginning of substance use’s impact on navigating challenges in care	A temporary escape from overwhelming negative emotions and loneliness
	Experiences of receiving care with varied levels of ability to treat patients with complex, unmet needs
Exploring current confined care and gratitude despite unease	Contrasting experiences with safety and punitive care while managing cravings in a restricted, confined environment
	Experiences with the patient–staff dynamic and its impacts on discrepancies and distance
Yearning for a somewhat normal life	Challenges and support-related needs to promote recovery
	Staying motivated to change one’s circumstances and lead a normal life

## Unveiling the beginning of substance use’s impact on navigating challenges in care

### A temporary escape from overwhelming negative emotions and loneliness

The first subcategory captured participants’ accounts of their initial experiences of experimenting with substances, why they began using them and the resulting effects. The participants often described unstable conditions at home with abuse as one of the forces that directed their life path towards using drugs. Two primary motivations emerged from the data, at least initially. First, the mood-altering effects of substances, including relieving feelings of anxiety and stress. As one participant explained, “When I tried it [using illicit drugs], I noticed how my anxiety subsided. I didn’t know what anxiety was, but I felt better with it. It slowly but surely led to more and more from there” (P7). The participants also narrated alleviating boredom or loneliness in life situations affected by social factors beset by life-challenging events. The use of drugs and/or alcohol became habitual and created a social connection to others in the same situation. Second, the risks of being involved in criminal activities were understood and perceived in a dual way among the participants. A clear line between their drug use and criminality was described; meanwhile, others did not recognize any connection between their criminal activities and SU: “There were a lot of benzodiazepines, maybe around 15. I think it was Clonazepam … more aggression than necessary, I believe. I used the wrong words and hurt him [a relative] more than necessary” (P3). Aggression and violence, in general, were described as not being a part of the participants’ self-views, but were instead connected to drug use and the associated psychotic breaks.

### Experiences of receiving care with varied levels of ability to meet patients with complex, unmet needs

Participants described experiences of being exposed to staff with a lack of knowledge and competencies while in a care setting. This meant that there was little support and sometimes also a total absence of individually tailored care plans. A desire for a specialized facility for SU integrated with treatment and care for mental health illness was expressed. This desire also included psychosocial support to increase their chances of a successful return to a life with no SU or criminality: “I can’t escape the fact that I was in a sensitive period, a sensitive age, and received the completely wrong help” (P8). Some participants expressed that, at times, their personal circumstances and timing prevented them from adhering to treatment. Mental health illness and the patients’ general health and life situations were so challenging sometimes that it became a barrier to receiving care. The caregivers failed to satisfy their needs in a holistic way due to the organizational structure and a mismatch between the content of the care and the patients’ factual needs: “Then I went to a counsellor. But there was so much going on around me. Anxieties … and I had completely given up hope on life. So, I didn’t go there anymore” (P7). Moreover, it was also noted how previous experiences impacted current trust and confidence during encounters with caregivers.

However, the participants also described positive experiences when they felt understood and included in their care: “They [staff] actually helped me to come to that conclusion, that I needed to go there. We gathered—the social worker, the addiction treatment centre staff and me—and we decided that it would be like that” (P6).

## Exploring confined care and gratitude despite unease

### Contrasting experiences with safety and punitive care while managing cravings in a restricted environment

Feelings of thankfulness for people who had prevented the continuation of a destructive life were stressed. The participants reflected on their feelings of safety in the FPC environment and with the staff therein. This was due to drug tests and safety controls performed in the units and routines that created an environment of safety and clear boundaries. However, feelings of worry and tension could also be present, as life outside the institution could sometimes follow them inside the institution. For example, they risked meeting “former enemies” in the same unit: “If you’ve lived in the world that I lived in … it could be an enemy, for example. Someone you have serious issues with. So, it’s an extra level of anxiety to know that someone can just appear” (P1).

The restricted environment in FPC was sometimes compared to prison. Being sentenced to care was described as entering a rigid environment with many rules that prompted feelings of being disconnected from their lives and being penalized by society for an uncertain length of time. Being cared for in FPC settings also created feelings of self-stigma and shame. One participant described deserving to be punished if they relapsed into SU: “I haven’t harmed anyone, but if I’ve taken something, I should just sit and feel ashamed, I think. And yeah, there’s not much more to say about it” (P4).

Participants explained how they struggled with triggers and cravings for drugs. Different strategies and behaviours were developed to withstand the cravings. Most participants had engaged in several parallel behaviours, such as consuming drinks high in caffeine, asking for medication as needed and overeating. In addition, the environment could entail many triggers, for example, aluminium foil from fast food or overhearing other inpatients talking about drugs. Other parallel behaviours that indicate psychological abstinence while being incarcerated in FPC were also described. These could include self-harm, getting involved in dangerous situations to trigger an adrenaline rush, drinking too fast (i.e., “sculling”), fantasizing about taking drugs, imagining visiting places where they used to consume drugs or alcohol, gambling, getting excited when nurses would draw medicine into syringes, engaging in excessive sexual fantasies or masturbation, being obsessed with a hobby and engaging in excessive physical activity.

### Experiences with unequal patient—staff dynamics and feelings of discrepancy and distance

Several participants described a lack of trust and a distance between themselves and staff, even though they did not have much contact with the staff, aside from addressing practical concerns. Patients’ experiences of insufficient care provision were related to a lack of staff who engaged in their care—that is, a lack of staff to interact with and who could activate them in their everyday living. At the same time, there was an understanding that one of the aims of FPC was to learn to tolerate boredom as a part of their treatment. In the data, an emerging discrepancy was observed between the participants’ self-perceptions of their psychiatric condition and the diagnosis that they had received for treatment, as well as regarding their prescribed medications. Some participants also emphasized not trusting other people in general and authorities in particular. They explained that they did not share the same views on or perceptions of society that most staff did, for example, with participants not sharing the belief that SU is a problem. This meant that there was a mismatch between the objective reality and their subjective understanding of their situation.

Moreover, they described a constant fear of being judged and of the risk assessments, which meant that it was subsequently a struggle to “fit” the expectations and be compliant with what was expected of them instead of allowing themselves to receive the care in a genuine way and become part of a therapeutic alliance and future re-orienting in life: “People come in here that you’ve never seen before…. You just have to display normal behaviour for them to be able to write normal behaviour. … Everyone has their own perception. It can go very wrong, you know” (P9).

The participants also described how their needs for care and treatment collided with the staff’s perceptions and assessments of their needs.

One participant voiced his experience as one of being punished for his behaviour with long-term consequences instead of getting the support that he felt he needed:

I had a bit of a problem in that my attention wore off quickly. So, I became impulsive and slammed a door, and I was punished severely, with Cisordinol [zuklopentixol] and Haldol [haloperidol]. … It caused a lot of tingling in my body, and I still have issues with that … because it dampens so much of the normality within me. (P5)

## Yearning for a somewhat normal life

### Challenges and support-related needs to promote recovery

This subcategory captures the needs emphasized by the participants due to SUD-related problems and the need for social connections and physical activities to encourage recovery and well-being. Receiving support from staff who were helpful and on whom they could rely was described as an important factor. The therapeutic relationship between staff and patients was understood as being genuine, straightforward, honest, firm, just, respectful and free from moral influence. Some participants appreciated staff members who provided information transparently:

I think one can be strict but fair. Or do you understand what I mean? Not that you can judge someone based on all the papers that are written; I don’t know how many papers are written about me every … every institution has something like that (P1).

Participants also mentioned the importance of being able to “read” the staff and reach out to specific individuals who made them feel secure. In addition, there seemed to be a lack of acknowledgement of the fact that many of the patients suffered from SUDs, and specific tools and discussions about how to handle SU-related problems were lacking. This resulted in inequality and injustice in care, as some patients were *invisible* to the staff, and some received the support they needed.

One participant noted a lack of discussion about SU, which he interpreted as an indication of not needing any treatment despite the struggle not to relapse. In contrast, others experienced challenges with relapses and described it as an automatic response that they could not preclude or say no to, although they were well aware of the consequences. Barriers to recovery included the complex nature of SU problems, where participants described uncertainty about what they needed: “It’s like pick-up sticks; you don’t know which stick to start picking up” (P1). Another barrier was a lack of confidence, as they did not dare to take the next step to engage in treatment.

General medication seemed to play a crucial role in helping participants feel normal and address their comorbid conditions:

It’s just as devastating as with drugs. When you become depressed, you don’t care about anything. … But with the right … levels of medication in me, I become free from depression and proper, just like I was in the beginning (P5).

A variation in medication, from sleeping pills to anti-psychotic medication, was seen as stable ground to be able to manage the struggle with SU issues. Many emphasized central stimulants as being essential for their well-being and as aids in ceasing drug use.

### Staying motivated to change one’s circumstances to lead a normal life

Participants provided examples of both their internal and external motivations to live a life less impacted by substances. Recovery was expressed as being akin to a change of circumstances, shifting into a new life and shaping a new identity. SUDs had prevented the participants from living, as they said, a *normal* life. SUDs had impeded their opportunities to work, have a driver’s licence, have a stable financial situation, stay housed and/or have children. Moreover, one barrier to starting a new life was expressed as difficulties with finding a new and meaningful social context: “It’s hard for me to think about what I should talk about, you know … yeah, everyone looks so proper, like … and then I come in” (P1).

Another barrier was the individual’s criminal/violent attachment. The participants’ own perceptions regarding the future risk of engaging in violence and criminal behaviour indicated their aim of transitioning away from a life of criminality:

When I’ve been around in criminal circles and such, I’ve had to struggle … I know deep down that you don’t do certain things. Committing crimes just makes me feel bad. The stress of stealing something or constantly worrying about getting caught: It’s tough. It’s better to live a peaceful life and obey the law. (P7)

Nevertheless, some motivations for discontinuing SU and expressions of hope for a better future with improved psychiatric and physical health were outlined in the data. To be able to reach patients, it seems highly relevant to explore their motivational level. Some participants wanted to live lives entirely free of drugs, others wanted to live their lives with weaker substances (e.g., switching from using opiates to using cannabis products instead), while some wanted a life with more sporadic SU.

## Discussion

In our study, we sought to explore Swedish FPC patients’ past and present experiences with, as well as future expectations of, their SU and SUD care. Overall, the findings indicate that the patients’ SUD experiences are inherently complex and often involved a lifelong struggle with various social and comorbid psychiatric problems, a circumstance that needs to be considered in SUD care in FPC.

The main theme of embracing harmony in opposition permeated all of the major categories and was considered to be central for staff to consider in offering treatment to the group of patients represented by the participants. This illustrates how such an understanding of patients’ perspectives on their experiences and ideas for the future generates important information on how to, as a care provider, facilitate the motivational process towards engaging in SUD treatment from the unique patient’s starting point, while at the same time navigating the coercive context of FPC.

Our results highlight important issues that care providers need to consider when formulating health plans for such a vulnerable subgroup within FPC, a patient group that requires specially adapted SUD care, both for their own well-being and to reduce the risk of committing future criminal acts (i.e., societal protection). The following discussion considers both the overarching structural conditions for SUD care within the FPC context, and the challenges associated with providing integrated SUD care in the FPC system.

## Forensic psychiatric care as a prerequisite for a better life

Although FPC involves a significant intrusion into personal integrity, patients can also experience it as being beneficial, at least to some extent. In our sample, some participants also expressed gratitude for coercive care, which they believed had protected them from relapsing and from potentially destructive situations in which they might have otherwise found themselves.

For FPC patients, the importance of feeling safe has been a recurring observation in many studies, especially if it encompasses protection from hostile individuals and/or engagement in the self-management of risks, so as not to jeopardize any privileges they have already gained (Senneseth et al., 2022; Shepherd et al., 2016). These results show that patients’ previous experiences are constantly present and need to be considered in their daily activities, as well as when planning for their lives after discharge from FPC. This aspect also connects with the high prevalence of trauma within addicted populations and the importance of trauma-informed approaches to help patients restore hope and meaning again (Missouridou, 2016).

Our findings show that participants appreciated or tolerated safety routines, such as inspections and mandatory drug tests, because those routines made them feel safer. Previous research has shown that patients can also perceive such routines as being difficult to comprehend, particularly in the initial stages following admittance, and that the actions of nurses and other healthcare professionals are seemingly arbitrary (Askola et al., 2018). This dynamic underscores the need for healthcare staff to be professional and to inform patients about both the practical aspects of their care (e.g., explanations of routines) and treatment-related aspects (e.g., explanations about medications and therapies). Such professionalism is important, as research has revealed that although patients who are involuntarily admitted generally perceive coercion as being unjustified, they might accept such coercion if the care is perceived as being necessary (Sugiura et al., 2020).

Our results additionally indicated participants’ desire for a new self-image and that changes in their circumstances could lead to more conventional, normal lives. For this reason, if staff provide patients with information on how SUD-related treatment is a necessary means to achieve such goals, then it could facilitate patients’ motivation to engage in treatment. Moreover, practical needs, such as stable housing, financial stability, engaging activities and forming new social connections were described as needing to be met. These findings align with previous results regarding FPC patients that highlight the importance of maintaining their hopes for a better future (Askola et al., 2018). Some patients in our study mentioned barriers and the need for help to overcome them, including difficulties with adjusting to new social settings and a distrust of authorities, which also aligns with past findings showing that situational factors related to increased support and supervision in everyday life are associated with a decreased likelihood of recidivism (Noland & Strandh, 2021).

During community reintegration, FPC patients also encounter discrimination, which, according to our results, was a factor when they considered the role of SU and SUDs in their futures. Although mental illness has been viewed with decreasing stigma over time, the same evolution has not been observed for SUDs (McFadden et al., 2022). FPC patients also have to grapple with their self-perceptions and how others perceive them as offenders. These factors can affect patients’ experiences of safety in FPC, as well as their perceptions of what their living conditions will be like after being discharged from FPC.

Our results additionally reveal patients’ desire for a different life and care staff’s need to meet patients where they are, without presupposing that they are fully aware of the consequences of SU in their lives, of them being unmotivated or of them lacking insight. One approach to engaging patients with lower levels of motivation or minimal goals of merely reducing SU can be, at least initially, to work from the perspective of harm reduction and minimize the worst consequences of SU, which requires a shift in perspective, but aligns with ideas in a Swedish governmental report concerning better treatment for SUDs (SOU 2023:62). This approach can be a first step towards further work, but it first has to be clarified in relation to the current regulations governing forensic psychiatry, which stipulate that FPC does not tolerate SU. The motivational interviewing framework is a valuable way to inform work on engaging patients’ interests in their treatment. However, because FPC patients are generally characterized as having complex mental health needs and low psychosocial functioning (Degl’innocenti et al., 2021), there is a need to develop new treatment approaches specifically for that group.

The currently inadequate treatment for patients with SUDs in FPC raises questions about evidence, staffing, knowledge of SU care and treatment, and structural limitations in integrating SUD treatment into clinical routines. Patients’ experiences with SUD care vary; some receive significant support from treatment programmes, whereas others are not asked about their SUDs. Because FPC traditionally focuses on treating primary diagnoses, such as psychosis, it often neglects comorbidities, such as SUDs (McFadden et al., 2022). Such neglect suggests that there is inequality in SUD treatment within FPC, possibly influenced by local structures and staffs’ interests and competence instead of individual patients’ needs.

## A treatment alliance as a prerequisite for integrated substance use disorder care in forensic psychiatric care

The patients in our study requested integrated treatment in which both their SUDs and comorbidities could be targeted within the same treatment programme, which is what research has shown to be the most efficient mode of treatment (Eagle et al., 2019). SUDs are complex and interact with various aspects of patients’ lives, especially in synergy with their psychiatric problems.

Our findings highlight the importance of recognizing that patients may hold conflicting views pertaining to several aspects of drugs and drug use, such as differences in views regarding the relationship between the effects of drugs and aggressive behaviour or criminality, as well as differences in ambitions regarding quitting drugs. It is therefore vital to explore each patient’s specific level of motivation (i.e., how urgent the patient perceives the need for treatment or change to be) while building an alliance with the patient to work with SU and uncover what kinds of outcomes the patient is motivated to achieve when forming a treatment alliance to overcome their SU.

A first step towards engaging patients is to show them from the very outset that staff are benevolently disposed and want to help, but that it takes time to develop trust. Forming a trustworthy relationship in which healthcare professionals can work with patients towards recovery requires patience and an empathetic understanding of the patient’s whole situation. As described in previous research, this process can take minutes or years, and a trustworthy therapeutic relationship can also contribute to the care environment being considered as caring instead of “locked” (Missouridou et al., 2022).

FPC patients with SUDs often have externalizing behavioural patterns that can be challenging for staff to handle in a professional manner. Such a heterogeneous patient group can also be affected both by different complex mental illnesses and by a distrust of authorities, which can consequently affect patients’ ability and motivation to engage in therapeutic relationships (McFadden et al., 2022). The participants also conveyed experiences of distance in their relationships with staff and discrepancies between the staff’s ideas and their own ideas about what constitutes appropriate care for various reasons. In turn, such distance and discrepancies could lead to frustration and feelings of powerlessness. When staff misuse their authority or behave in a way that is perceived to be unfair or inconsistent, it only heightens the sense of distrust and detachment among patients towards the staff (Livingston et al., 2013; Nyman et al., 2022; Söderberg et al., 2022). The same research has also described how this impacts the openness of patients in their narratives, for example, by them refraining from sharing personal information and avoiding forming relationships with staff. What is evident from our interviews is that a major impediment to personal recovery is feelings of isolation and powerlessness. Thus, improving meaningful connections between patients and staff, actively involving patients in discussions about their treatment and finding at least some common ground when establishing treatment goals together are essential (Senneseth et al., 2022). Another component in the recovery process is to show that the patient is trusted, which can also influence their self-determination and self-confidence (Mangoulia et al., 2021). Our results highlight the importance of respecting patients’ perceptions of important past and present events in their lives and care, as well as their motivation, all of which has impacted their history with SUDs and their subjective views on SU. Patients have had many care- and SUD-specific experiences that are connected in various ways before entering FPC. The consideration of each patient’s history is central to the chief tenets of PCC. Following a PCC approach means acknowledging the importance of patients’ narratives and striving to develop collaborative patient—professional partnerships (Ekman & Swedberg, 2022). The participants in our study emphasized transparent communication as being valuable as well. Indeed, being transparent when communicating with patients and explaining the ways in which patients can have full influence, engage in shared decision-making or have no influence can characterize a professional approach in FPC contexts (Söderberg et al., 2022).

The FPC patients with SUDs in our study also described a trustworthy relationship with staff as being genuine, straightforward, just, firm, respectful and free from moral influence, aspects that have also been highlighted in previous studies by FPC patients in general (Livingston et al., 2013; Söderberg et al., 2022). As described by Nyman et al. (2022), FPC participants have reported feeling punished due both to their sentencing and to occasional punitive measures during FPC. Therefore, it is crucial for staff to communicate clearly to prevent misunderstandings and build trust, as emphasized in the interviews in our study. SU is considered a clinical risk factor for violence, one that leads to the revocation of privileges following relapse. At the same time, previous research has shown that a penalizing and consequential attitude among some FPC staff exacerbates patients’ views that their treatment is punitive (Green et al., 2023). A proactive, collaborative approach, including personalized communication, might improve patients’ understanding of restrictions, enhance staff awareness of their impact and lay the groundwork for future discussions on managing potential responses in the future (Marklund et al., 2020).

Most patients in our study articulated the need for medication to manage their psychiatric comorbidities as a prerequisite for engaging in work concerning their SU, such as the need for medication to address psychosis or depression. However, other patients expressed a lack of understanding about their diagnosis and disagreement with the staff regarding the need for medication, such as anti-psychotic drugs. In FPC, patients are often subjected to polypharmacy, which makes discerning the effects of individual pharmaceuticals challenging (Howner et al., 2020). Several patients also expressed how central stimulant therapy was imperative for effectively managing their daily lives and how they no longer needed to seek out illegal substances or circumvent the onset of drug cravings. However, from an FPC perspective, there are some problems with such an understanding. First, there is no approved pharmacologic treatment aimed at treating amphetamine use disorder, and second, within FPC, physicians have to also consider the risk of induced psychosis or other psychiatric side effects related to central stimulants, including the possibility of increased drug cravings. Furthermore, there is no consensus among forensic psychiatric clinics in Sweden regarding central stimulants, and prescription practices vary.

## Strengths and limitations

When selecting quotations from the interview transcripts that represented meaning units related to the study’s aim, two of the authors—the interviewer (i.e., CS) and a co-author (i.e., CH)—independently chose statements from the first two transcripts. Upon comparison, it was found that they had selected similar meaning units, which enhanced the credibility of the analytical process (Korstjens & Moser, 2018). Another strength of our study was that it provided insights into an unusually hard-to-reach patient group and it enabled us to explore their experiences. Moreover, when coupled with the interviewer’s experience with the context, it allowed for relevant follow-up questions to be posed. Another strength was that all five of the authors had different theoretical and professional perspectives and knowledge about the subject as mental health nurses and psychological and medical health professionals.

All participants had experiences of being treated in the same forensic psychiatric hospital and suffered in different ways from SUDs. There was, however, diversity in terms of age, diagnoses and functional levels across the various units, which enhances our study’s transferability (Graneheim et al., 2017). At the same time, age, diagnosis and length of stay were self-reported and thus presented the risk that those facts were not exact. Despite potential influences from past SU and severe mental illness, the participants demonstrated varying abilities to discuss their experiences, but they all contributed unique perspectives on different aspects of SU and SUD care. As such, the totality of the data was considered to be sufficient to achieve the study’s aim (Vasileiou et al., 2018). The participants’ characteristics and knowledge of SU and comorbidities were specific to that aim, and their diverse experiences have not formerly been sufficiently described, which Malterud et al. (2016) has described as a part of information power requiring fewer participants. The data gathered in our study was rich, as participants’ experiences confirmed each other’s experiences and captured the phenomenon in terms of a long history with SUD, psychiatric illness and FPC. Although some participants even experienced delusions and paranoid thoughts due to their condition, it is nevertheless valuable and ethically important to document those experiences, as healthcare professionals have to engage with patients in their current mental state.

The absence of female participants in our study might limit its transferability. Even so, the gender imbalance reflects the larger population under consideration, and our results align with past findings from studies that only included women (Revelj et al., 2023). In future research, it is therefore crucial to attempt to recruit females as well as males. Interviewing participants under compulsory care who suffer from severe mental illness is a complex process. The first author (i.e., CS) works as a clinician in the units and is known by many of the patients, although she is not formally responsible for their care or treatment. This dynamic may have influenced the interview process and, in turn, the results. However, acknowledging preunderstandings also enhances prolonged engagement and understanding of the context, which is considered a strategy for ensuring the trustworthiness of the results (Korstjens & Moser, 2018).

Research has suggested the potential risk of participants feeling ashamed about relapses and cravings, thereby leading them to withhold information to avoid disappointing their caregivers (Green et al., 2023). This dynamic could result in patients being less motivated, declining participation in the study and biasing the results. On that point, total absolutism was not entirely endorsed by the participants, thereby indicating that they had at least a certain amount of freedom to express themselves (i.e., because SU would be deemed illegal).

## Clinical implications and directions for future research

Our findings reveal an urgent need for FPC to provide equal SUD care that is implemented at the national level, that is not dependent on local resources and that has a clear policy whereby, in FPC, every patient should undergo a specific structurally integrated SUD, comorbidity and aggressive behaviour anamnesis. First, patients need to be presented with feasible opportunities to actively participate in their care. Second, patients also need to be informed in a transparent, nonjudgmental way, one adapted to their level of cognitive functioning, about the effects, risks and harms of SU and how substances can increase the risk of violence and impaired comorbid psychiatric or physical illness, as also recommended in Swedish governmental report: we can do better (SOU, 2023: 62). Collaboratively developing health plans for handling post-relapse scenarios can reduce patients’ perceptions of being punished and staff’s penalizing attitudes. This method of care provision requires further evaluation in subsequent research studies.

In the future, staff and patients should work together to document the patients’ needs and create tailored interventions in personalized health plans. This process should also involve outlining the individual’s personalized goal(s) for engaging in SU treatment, such as relapse prevention or harm reduction. More research is also needed to develop treatments customized to individuals with thought disturbances or cognitive disabilities.

Given the considerable prevalence of SU and SUDs for FPC patients, educational interventions aimed at healthcare personnel directly involved in daily patient care, including nurses, are necessary and should address the topic of detecting parallel behaviour. Moreover, due to the complexity of treating both SUD and comorbid psychiatric illness, it is still necessary for supervisors or possibly staff to consult experts for support regarding how to provide individualized care and keep the implementation of care sustainable.

## Conclusion

Our study has revealed the subjective perspectives of FPC patients with SUDs and their need for a safe environment that enables recovery. This includes their life stories and hopes for a better future. Healthcare organizations where FPC is in place must recognize the significance of individually tailored treatment/care plans and programmes for SU to support the successful (re)habilitation of these patients. SU is an increasing challenge in FPC, and its consequences for individuals, their loved ones and society need to be addressed. FPC aims not only to confine individuals who have committed a crime while in the grips of a serious mental illness, but is also responsible for providing a recovery-oriented practice characterized by trust, genuineness and fairness.

## Supplementary Material

interview guide.docx

## Data Availability

The datasets generated and analysed during the study are not publicly available due to their sensitive nature (e.g., containing personal accounts that could be linked to a specific individual). They are nevertheless available from the corresponding author upon reasonable request and under certain circumstances.

## Submission declaration and verification

This study has not been published previously, it is not under consideration for publication elsewhere, publication is approved by all authors and tacitly or explicitly by the responsible authorities where the work was carried out, and, if accepted, it will not be published elsewhere in the same form, in English or in any other language, including electronically, without the written consent of the copyright holder.
