# Supplementary material for: Gratitude despite unease among Swedish male forensic psychiatric patients with substance use disorders: an interview study
Source: Int J Qual Stud Health Well-being. 2024 Nov 2;19(1):2418671. doi: 10.1080/17482631.2024.2418671 (PMC11536691; doi:10.1080/17482631.2024.2418671)
Supplement: interview guide.docx [file ZQHW_A_2418671_SM3933.docx]

**Semi-Structured Interview Guide**

*Introduction and Information*

Thank you for participating. Your thoughts and experiences are crucial for us to better understand and improve substance use care. The questions will revolve around your experiences with alcohol/drugs, the treatments you've received, your opinions on them, and what you believe could help you quit.

You have the right not to answer questions if you prefer and are not obligated to provide a reason.

You can end the interview whenever you wish.

The interview will be recorded.

Do you have any questions about what I've explained?

Is it okay if we begin?

**DUDIT-E / AUDIT – E**

*Given to the patient for self-assessment – the interviewer remains in the room.*

We can start with some background questions.

How old are you?

Which diagnoses do you have?

How long have you been convicted to forensic psychiatric care?

**Past Experiences – Motivation, Capacity, Barriers**

- 1. *What prompted you to start using alcohol/drugs?*
- For example: How did alcohol/drugs impact you?
- Can you describe a situation in your life when you searched help for quit using alcohol/drugs? For example: social relationships, living situation, financial situation, any specific event
  1. *Can you tell me about a time when you sought help to quit alcohol/drug use?*
- For example: emergency room, outpatient care, social services
- For example: Experiences and insights, personal expectations, type of care/treatment, motivation, treatment effectiveness

**Present Motivation, Barriers, Interaction, Stigmatization**

Now, I would like to ask about your current situation.

2.1. *What does a typical day look like in here?*

- For example: security, rules and controls, whether you chose to be here

2.2. *Would you describe, from start to finish, a conversation you had about alcohol/drugs with FPC staff?*

- For example: during a relapse, experience of staff understanding when you ask for help, trust from staff, is there reasonable expectations on being drug-free
- How could the staff have said/done/helped better during discussions/interventions about drugs/alcohol?
- What would you say to yourself if you were the staff?

2.3. *Do you think about alcohol/drugs as much here as you did before coming here?*

- For example: How often do you have these thoughts, what do you do when you think about or crave alcohol/drugs on the ward

**Future Motivation, Capacity**

Now, let's talk about how you view your future.

3.1. *If you had complete control, how would you like to use alcohol/drugs in the future?*

- For example: If you want to reduce, what kind of help, how? Is it the responsibility of the care system or yourself? Your own motivation?

3.2. *You mentioned earlier that you were influenced by drugs. Had you used alcohol/drugs the day before or on the same day as the incident that brought you here?*

- For example: What effects, anxiety, hangover, future risk of violence

3.3. *Is there anything important you would like to add?*

Follow-up questions will be asked as needed, for example:

- Can you elaborate on what you mean?
- Do you have an example?
- What did you feel then?
- How did you perceive it?
- What do you think it is due to?
